# Heterogeneity Signs on Noncontrast Computed Tomography Predict Hematoma Expansion after Intracerebral Hemorrhage: A Meta-Analysis

**DOI:** 10.1155/2018/6038193

**Published:** 2018-01-10

**Authors:** Danfeng Zhang, Jigang Chen, Qiang Xue, Bingying Du, Ya Li, Tao Chen, Ying Jiang, Lijun Hou, Yan Dong, Junyu Wang

**Affiliations:** ^1^Department of Neurosurgery, Changzheng Hospital, Second Military Medical University, Shanghai 200003, China; ^2^Department of Neurology, Changhai Hospital, Second Millitary Medical University, Shanghai, China; ^3^Force 71571, PLA, China

## Abstract

**Background and Purpose:**

Hematoma expansion (HE) is related to clinical deterioration after intracerebral hemorrhage (ICH) and noncontrast computed tomography (NCCT) signs are indicated as predictors for HE but with inconsistent conclusions. We aim to clarify the correlations of NCCT heterogeneity signs with HE by meta-analysis of related studies.

**Methods:**

PubMed, Embase, and Cochrane library were searched for eligible studies exploring the relationships between NCCT heterogeneity signs (hypodensity, mixed density, swirl sign, blend sign, and black hole sign) and HE. Poor outcome and mortality were considered as secondary outcomes. Odds ratio (OR) and its 95% confidence intervals (CIs) were selected as the effect size and combined using random effects model.

**Results:**

Fourteen studies were included, involving 3240 participants and 435 HEs. The summary results suggested statistically significant correlations of heterogeneity signs with HE (OR, 5.17; 95% CI, 3.72–7.19, *P* < 0.001), poor outcome (OR, 3.60; 95% CI, 1.98–6.54, *P* < 0.001), and mortality (OR, 4.64; 95%, 2.96–7.27, *P* < 0.001).

**Conclusions:**

Our findings suggested that hematoma heterogeneity signs on NCCT were positively associated with the increased risk of HE, poor outcome, and mortality rate in ICH.

## 1. Introduction

Intracerebral hemorrhage (ICH) accounts for nearly 15% of the stroke and is usually regarded as the most deadly type [1, 2]. It carries a poor prognosis, with 40% mortality at one month and only 20% survivors being independent at six months [3]. Initial volume, shape, and location of hematoma are reliable predictors of mortality and clinical functional outcomes [1, 4]. However, these are unmodifiable factors on admission. Hematoma expansion (HE) is usually considered as hemorrhage volume growth of more than 12.5 mL or 33% on repeated head computed tomography (CT) and occurs in almost one-third of ICH patients [5]. As a modifiable factor, it has been regarded as an independent predictor for unpleasant outcomes and mortality after ICH [6–8].

The spot sign, an area of contrast enhancement visible on computed tomographic angiography (CTA), is a well-established factor which predicts HE in ICH patients [9, 10], and therefore it carries potential clinical implications in identifying patients at a high risk of early death and poor functional outcomes. However, identification of spot sign needs a CTA, which is much more expensive and time-consuming than regular CT scan and not routinely conducted at acute phase in many clinical settings.

In contrast, as a preferred imaging test for ICH patients, noncontrast computed tomography (NCCT) is widely available due to its low price and convenience. Previous studies have examined the accuracy of NCCT signs such as hypodensity, mixed density, swirl sign, blend sign, and black hole sign in predicting HE, but these conclusions are inconsistent based on different methods and subjects [11–16]. A better understanding of NCCT signs might help identify patients with stratified needs and potentially improve prognosis. We carry out this study to evaluate the correlations of NCCT heterogeneity signs with HE after ICH.

## 2. Methods

### 2.1. Search Strategy

Our meta-analysis was reported according to the recommendations of Preferred Reporting Items for Systematic Reviews and Meta-Analysis: The PRISMA Statement [17]. The protocol was not previously registered. Two authors (D.F. Z. and J.G. C.) searched PubMed, Embase, and Cochrane library independently for eligible studies exploring the relationships between heterogeneity signs on NCCT and HE on July 15th 2017 with no date limits. The language was limited to English. Terms “hypodensity”, “mixed density”, “swirl sign”, “blend sign”, and “black hole sign” were combined with free words “intracerebral hemorrhage” and “computed tomography” in retrieval. The search strategy for PubMed was available in online-only Data Supplement. The references of included articles were also examined.

### 2.2. Heterogeneity Signs and Endpoints

Available heterogeneity signs included in our study were hypodensity, mixed density, swirl sign, blend sign, and black hole sign on NCCT. According to previous studies, hypodensity, mixed density, and swirl sign on NCCT were defined as hypoattenuated or isoattenuated region(s) within hyperattenuated ICH, while blend sign or black hole sign was considered as the combination of relatively hypoattenuating region and hyperattenuating area with a well-defined margin [16, 19–23]. All these definitions indicated hypoattenuated region(s) within hyperattenuated ICH, and thus we used terms heterogeneity signs to represent hypodensity, mixed density, swirl sign, blend sign, and black hole sign on NCCT in current study.

Our endpoints were considered to be primary and secondary. The primary endpoint was HE and defined as a hematoma growth of “>6 mL or >33%” or “>12.5 mL or >33%.” The secondary endpoints were poor outcome and mortality (in-hospital or 90-day mortality). Poor outcome was defined as Glasgow Outcome Scale (GOS) score of 1 to 3, or modified Rankin scale (mRS) score of more than 3, or Glasgow Come Scale (GCS) score of less than 8, or decreased GCS score of more than 3.

### 2.3. Inclusion Criteria

Studies were included in our research if they (1) were case control or cohort studies; (2) recruited patients suffering ICH; (3) examined the associations between the presence of heterogeneity signs and primary or secondary endpoints. Studies with incomplete data were excluded and in the evaluation of heterogeneity signs; we considered hypodensity as the exposure factor if several NCCT signs were available in one study.

### 2.4. Data Extraction and Quality Assessment

Characteristics and data were extracted from included studies independently by three investigators (D.F. Z., J.G. C., and Y. L.), which were as follows: first author, year of publication, study design, study population, sample size, age and gender of subjects, time interval from ICH onset to CT, baseline GCS scores, definitions of heterogeneity signs and endpoints, and number of participants with or without events in heterogeneity-signs-positive and negative groups. Two authors (T. C., Y. D.) independently assessed methodological quality using the Newcastle-Ottawa Scale. Discrepancies among investigators were resolved by joint review.

### 2.5. Statistical Analysis

Dichotomous data were available for the meta-analysis. Odds ratio (OR) and its 95% confidence intervals (CIs) were selected as the effect size. Heterogeneity among studies was assessed with the *I*^2^ statistic and Chi-square test. *I*^2^ values of 25%, 50%, and 75% were considered as low, moderate, and high, respectively [24]. Random effects model was employed in all quantitative analysis. Subgroup analyses were conducted according to study design, sample size, mechanism of ICH, and time interval from symptom onset to CT. Sensitivity analyses were conducted by removing one study at a time. Publication bias was assessed with Egger's test. A significant level of *P* = 0.1 was used in the evaluation of heterogeneity and publication bias. In other cases, *P* value of less than 0.05 was deemed as significant. All statistical analyses were conducted using STATA software (version 12.0; Stata Corporation, College Station, TX).

## 3. Results

### 3.1. Literature Search

**Figure 1:**
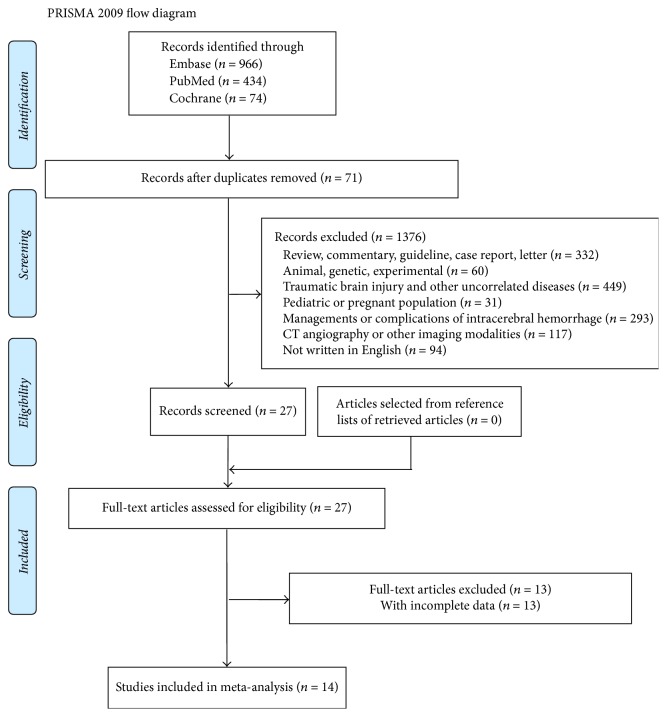
The flow diagram of the search process, from [18]. For more information, visit http://www.prisma-statement.org.

The study-retrieval process was shown in Figure 1. The initial search produced 966 studies from Embase, 434 studies from PubMed and 74 studies from Cochrane library. After the removal of duplicates and irrelated publications, 27 studies were potentially related to our review and the full texts were assessed. Finally, a total of 14 articles were included in our analysis. The review of reference lists of included studies yielded no eligible literatures.

### 3.2. Study Characteristics

Fourteen studies were included in the meta-analysis, which consisted of one prospective cohort study, three retrospective cohort studies, and ten case control studies, involving 3240 participants and 435 HEs (Table 1 and Supplementary Table I). Five studies were conducted in North America [11, 15, 21, 25, 26], four in Europe [13, 19, 22, 27], and five in Asia [16, 20, 23, 28, 29]. Generally, the gender ratio was balanced except in Pruthi et al., where males account for 91.7% of sample size. Presence of hypodensities was reported in two studies [21, 26], with mixed density reported in two studies [15, 16], swirl sign in six studies [11, 13, 19, 21, 25, 27], blend sign in four studies [20–22, 30], and black hole sign in two studies [23, 28]. One study reported several NCCT signs for the same cohort [21]. Definitions of heterogeneity signs and quality assessment of included studies were demonstrated in Tables I and II in the online-only Data Supplement.

### 3.3. Heterogeneity Signs and HE

**Figure 2:**
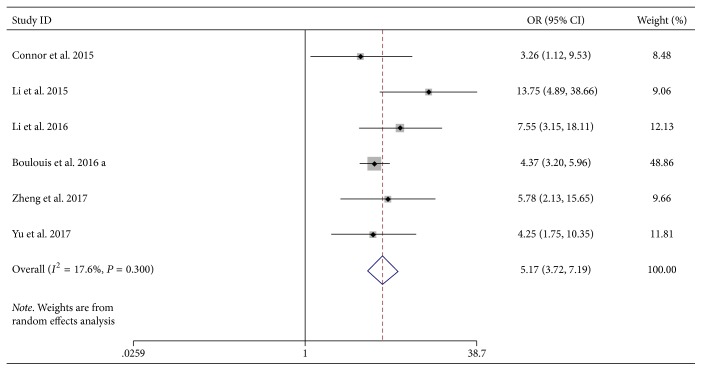
Forest plots of NCCT heterogeneity signs and HE. CI, confidence interval; HE, hematoma expansion; NCCT, noncontrast computed tomography; OR, odds ratio.

Six studies were available to investigate the relationship between NCCT heterogeneity signs and HE [20, 21, 23, 25, 28, 29]. The summary OR suggested statistically significant relationship between heterogeneity signs and HE (OR, 5.17; 95% CI, 3.72–7.19, *P* < 0.001; Figure 2) with little evidence of heterogeneity (*I*^2^ = 17.6%, *P* = 0.30).

### 3.4. Heterogeneity Signs and Poor Outcome

**Figure 3:**
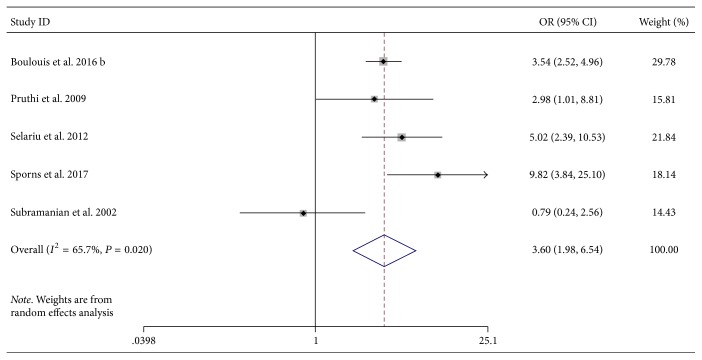
Forest plots of NCCT heterogeneity signs and poor outcome. CI, confidence interval; HE, hematoma expansion; NCCT, noncontrast computed tomography; OR, odds ratio.

Five studies explored possible associations between heterogeneity signs on NCCT and poor outcomes [15, 16, 19, 22, 26]. Overall, we detected a significant increased incidence of poor outcome among patients with heterogeneity signs (OR, 3.60; 95% CI, 1.98–6.54, *P* < 0.001, Figure 3) with moderate heterogeneity (*I*^2^ = 65.7%, *P* = 0.02).

### 3.5. Heterogeneity Signs and Mortality

**Figure 4:**
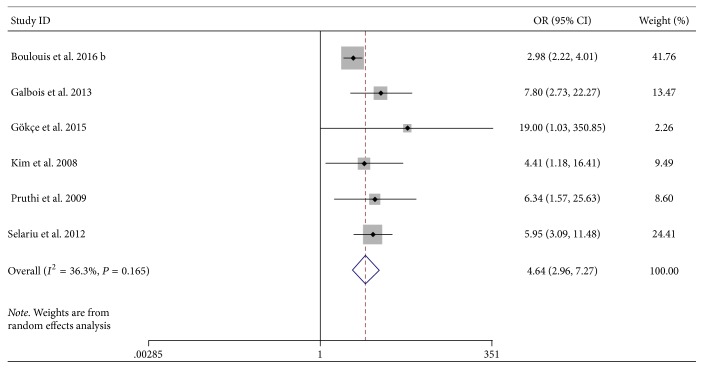
Forest plots of NCCT heterogeneity signs and mortality. CI, confidence interval; HE, hematoma expansion; NCCT, noncontrast computed tomography; OR, odds ratio.

Six studies were included to evaluate the relationship between NCCT heterogeneity signs and mortality [11, 13, 16, 19, 26, 27]. The pooled result favored a positive association between NCCT heterogeneity signs and mortality (OR, 4.64; 95% CI, 2.96–7.27, *P* < 0.001, Figure 4) with low heterogeneity (*I*^2^ = 36.3%, *P* = 0.17).

### 3.6. Subgroup Analysis, Sensitivity Analysis, and Publication Bias

Results of subgroup analyses were available in Table III in the online-only Data Supplement. In the analysis of HE and mortality, differences in all subgroups were statistically significant, while no significant results were detected in the subgroups of sample size < 150 and secondary ICH (*P* = 0.1) when analyzing the relationship between heterogeneity signs and poor outcome. In the sensitivity analysis, no significant result was detected when excluding studies one by one in all analysis. Publication bias was not detected by Egger's test except in analysis examining the relationship between heterogeneity signs and mortality (*P* = 0.031).

## 4. Discussion

Significant HE was a well-established predictor of clinical prognosis and mortality in ICH, and NCCT findings might potentially predict HE as suggested by previous studies [6–8]. Our results revealed that heterogeneity signs detected on NCCT were positively associated with the increased risk of HE (*P* < 0.001), poor outcome (*P* < 0.001), and mortality rate (*P* < 0.001), indicating the potential predictive value of heterogeneity signs for HE, poor prognosis, and mortality in ICH patients. In the subgroup analysis of poor outcome, there were no statistically significant differences for the subgroups of sample size < 150 and secondary ICH, which might imply that small sample size or secondary ICH would increase the heterogeneity and undermine the predictive value of heterogeneity signs in ICH.

The pathophysiology of NCCT heterogeneity remains elusive currently. Hematoma density on NCCT has close relationship with the phase of hemorrhage, number of foci of the blood, and hematocrit [31, 32]. It is suggested that short time interval to NCCT, large hematoma volumes, and anticoagulants are all related to NCCT heterogeneity, as well as to severe clinical presentations and worse prognosis [33]. Pathophysiologically, hypodensities on NCCT seem to indicate blood at an early evolution stage [21, 31]. Liquid blood tends to hypoattenuate on NCCT relative to surrounding structures in acute phase [31]. However, whether hypodensity implies active hemorrhage or just responds to impaired coagulation processes is still unknown [34].

According to our study, heterogeneity signs included a broad spectrum of imaging markers on NCCT, among which the hypodensity [21, 35], swirl sign [21, 25], blend sign [20, 21, 29, 35], and black hole sign [23, 28] had been investigated previously regarding their relationships with hematoma growth. Hypodensities were proved to be associated with HE in two studies conducted by Boulouis et al. and Morotti et al. [21, 35]. Similarly, swirl sign and black hole sign were also indicated to be positively related to HE [21, 23, 25, 28]. However, four studies explored the predictive value of blend sign for HE and their conclusions were different. As for Li et al., Zheng et al., and Morotti et al., they detected that blend sign was a predictive factor for hematoma growth [20, 29, 35], while this relationship was not statistically significant in the study by Boulouis et al. [21].

Additionally, previous studies explored the relationships of NCCT markers including hypodensities [26, 35], mixed density [15, 16], swirl sign [11, 13, 19, 27], and blend sign [22, 35] with clinical outcome after ICH. Swirl sign and blend sign were indicated to be statistically associated with clinical outcome such as unfavorable outcome, mortality, or brain death [11, 13, 19, 22, 27, 35]. However, the conclusions were inconsistent concerning the predictive value of hypodensities and mixed density for clinical outcome. Morotti et al. failed to detect a positive connection between hypodensities and unfavorable outcome in the multivariate analysis [35]. Subramanian et al. [15] proved in a case control study involving 51 participants that NCCT mixed density was not correlated with the increased risk of active bleeding or unfavorable outcome. The discrepancies might derive from the inherent interpreter differences in identifying hematoma hypodensity or variability in recording active bleeding. Therefore, the assessment of density alone should not be applied in routine clinical practice and more researches were needed regarding the optimal method of evaluating density or predicting HE [14].

To our knowledge, this study was the first systematic review and meta-analysis available exploring the relationships between heterogeneity signs on NCCT and the presence of HE. Our results supported that hematoma heterogeneity signs were potential predictors in identifying patients at a high risk of HE. In addition, we investigated the relationships between hematoma heterogeneity signs and prognosis of ICH, and statistically significant results were detected. Our study also had advantage in including trials from different countries and regions which were representative enough. These included studies were all newly published in recent years and the heterogeneity among studies was moderate.

Several limitations in our study should be noticed. Firstly, relationships between hematoma heterogeneity and HE or clinical outcome were not examined due to the differences between the definitions of hematoma heterogeneity and NCCT signs in our study. Hematoma heterogeneity was defined according to a 5-point grading scale first introduced and validated by Barras et al. [12], as density categories of no or small heterogeneity (1 or 2) were labeled homogeneous and categories of progressive heterogeneity (3 to 5) heterogeneous. However, there were overlaps between hematoma heterogeneity and heterogeneity signs, and thus some valuable patient data might be missed in our study. Secondly, there was variability among included studies regarding the definition of heterogeneity signs and HE, which might derive from the different criteria used in different studies. Thirdly, despite moderate heterogeneity in our analysis, different biases such as the selection bias or publication bias did exist owing to the defects of meta-analysis itself. Patients with NCCT heterogeneity signs might have large initial volume or irregular shape of hematoma [12]. The language was confined to English, which might overlook some valuable non-English studies. Notably, we found publication bias in the analysis of mortality, indicating a potentially overestimated association between NCCT heterogeneity signs and mortality, since positive results were more likely to be reported. Fourthly, different indicators with variable definitions were used to evaluate the clinical outcomes in included literatures, which might be the source of heterogeneity among studies [26, 36]. Lastly, the quantity of included studies was limited and most of them were retrospective, without eligible high-quality trials.

## 5. Conclusions

Despite the limitations, our findings raised certain clinical implications that hematoma heterogeneity signs were positively associated with an increased risk of HE and poor clinical outcome and mortality rate in ICH. However, caution was needed when interpreting our results due to the moderate heterogeneity among studies and limited number of studies in our analysis. Further large scale prospective study was required to confirm these findings in the future.

## Figures and Tables

**Table 1:** Characteristics of included studies.

Author(s), year	Study design	Study population	Sample size, men (%)	Age (years)	Mechanism of ICH	Exposures	Outcomes	Definition of HE or clinical outcome	Time interval from symptom onset to CT (hours)	Baseline GCS score
Subramanian et al., 2002	Case control	USA	51, NA	NA	Traumatic hemorrhage	Mixed density	Poor outcome	GOS score of 1–3	NA	NA

Kim et al., 2008	Case control	USA	56, 62.5	Mean: 62.8	Primary ICH	Swirl sign	Hospital mortality	Death before discharge	Median, IQR: 13, 7.3–25.5	Mean: 11.6

Pruthi et al., 2009	Case control	India	109, 91.7	Mean: 31	Traumatic or spontaneous ICH	Mixed density	Poor outcome, mortality	GCS score of ≤8 at discharge, death at discharge	NA	NA

Selariu et al., 2012	Case control	Sweden	203, 44.8	Mean, SD: 73, 14	Spontaneous ICH	Swirl sign	Unfavorable outcome, mortality	mRS of ≥4 at three-month follow-up, death at three-month follow-up	On admission	NA

Galbois et al., 2013	Case control	France	72, 56.9	Mean: 71.5	Spontaneous ICH	Swirl sign	Brain death	(i) Absence of consciousness; (ii) abolition of all brainstem reflexes; (iii) loss of bioelectrical activity	Median, IQR: 2; 2-3	Median, IQR: 7, 5–11

Gökçe et al., 2015	Case control	Turkey	45, 55.6	Mean, SD: 68.8, 10.8	OAC-associated ICH	Swirl sign	Mortality	In-hospital death	On admission	NA

Connor et al., 2015	Case control	Canada	71, 65	Mean, SD: 68.2, 15.6	Primary ICH	Swirl sign	HE	≥6 ml or ≥33% on follow-up CT	Median, IQR: 1.9, 1.3–4.6	Baseline NIHSS: median, IQR: 10, 6–16

Li et al., 2015	Case control	China	172, 68	Mean, SD: 61, 12	Spontaneous ICH	Blend sign	HE	>12.5 mL or >33% on follow-up CT	Within 6 hours	NA

Boulouis et al., 2016^a^	Retrospective cohort	USA	1029, 55	Mean: 71.8	Primary ICH	Hypodensities, swirl sign, blend sign	HE	>6 mL or >33% on follow-up CT	Median, IQR: 4.9, 2.5–8.1	Median, IQR: 12, 7–15 (hypodensity); 14, 10–15 (no hypodensity)

Boulouis et al., 2016^b^	Retrospective cohort	USA	800, 55.8	Mean: 72	Primary ICH	Hypodensities	Unfavorable outcome, death	mRS > 3 at 90 days	Within 24 hours	Mean: 12.1

Li et al., 2016	Prospective cohort	China	206, 65.5	Mean, SD: 60.3, 12.2	Spontaneous ICH	Black hole sign	HE	>12.5 mL or >33% on follow-up CT	Within 6 hours	Black hole sign positive: mean, SD: 10.6, 3.7; Black hole sign negative: mean, SD: 12.4, 3.1

Sporns et al., 2017	Case control	Germany	182, 54.4	Median, range: 68, 54–79	Spontaneous ICH	Blend sign	Poor outcome	(1) Early hemicraniectomy (2) decreased GCS score of >3 within 48 hours	Within 6 hours	Median, IQR: 11.5, 10–13

Yu et al., 2017	Case control	China	129, 72.9	Mean, SD: 59, 11.6	Spontaneous ICH	Black hole sign	HE	>12.5 mL or >33% on follow-up CT	Within 6 hours	NA

Zheng et al., 2017	Retrospective cohort	China	115, 73	Mean, SD: 58.8, 11.6	Spontaneous ICH	Blend sign	HE	>12.5 mL or >33% on follow-up CT	Within 6 hours	NA

CT, computed tomography; GCS, Glasgow coma scale; GOS, Glasgow outcome scale; HE, hematoma expansion; ICH, intracerebral hemorrhage; IQR, interquartile range; mRS, modified Rankin scale; NA, not available; NCCT, noncontrast computed tomography; NIHSS, national institute of health stroke scale; OAC, oral anticoagulant; SD, standard deviation.

## Supplementary Materials

Supplementary Materials
*Supplemental Method.* The complete search algorithm for PubMed is as follows. PubMed ((((((((((intracerebral hemorrhage) OR intracranial hemorrhage) OR intracerebral hematoma) OR intracranial hematoma) OR cerebral hemorrhage) OR subdural hemorrhage) OR epidural hemorrhage)) AND (((((hypodensit^*∗*^) OR density) OR swirl sign) OR blend sign) OR black hole sign))) AND computed tomograph^*∗*^* Supplemental Table I*. Categorization, exposure, and original data of included studies. CT, computed tomography; HE, hematoma expansion; HU, Hounsfield unit; ICH, intracerebral hemorrhage;* N*, number.* Supplemental Table II*. Quality scores of included studies using Newcastle-Ottawa Scale (maximum score of 9). 0 = “No,” “Unable to determine,” or “Not available”.* Supplemental Table III*. Subgroup analyses of heterogeneity signs on NCCT and risk of HE. CI, confidence interval; HE, hematoma expansion; ICH, intracerebral hemorrhage; NCCT, noncontrast computed tomography; OR, odds ratio.
