# Supplementary material for: Heterogeneity Signs on Noncontrast Computed Tomography Predict Hematoma Expansion after Intracerebral Hemorrhage: A Meta-Analysis
Source: Biomed Res Int. 2018 Jan 10;2018:6038193. doi: 10.1155/2018/6038193 (PMC5818889; doi:10.1155/2018/6038193)
Supplement: Supplementary Materials — Supplemental Method. The complete search algorithm for PubMed is as follows. PubMed ((((((((((intracerebral hemorrhage) OR intracranial hemorrhage) OR intracerebral hematoma) OR intracranial hematoma) OR cerebral hemorrhage) OR subdural hemorrhage) OR epidural hemorrhage)) AND (((((hypodensit∗) OR density) OR swirl sign) OR blend sign) OR black hole sign))) AND computed tomograph∗ Supplemental Table I. Categorization, exposure, and original data of included studies. CT, computed tomography; HE, hematoma expansion; HU, Hounsfield unit; ICH, intracerebral hemorrhage; N, number. Supplemental Table II. Quality scores of included studies using Newcastle-Ottawa Scale (maximum score of 9). 0 = “No,” “Unable to determine,” or “Not available”. Supplemental Table III. Subgroup analyses of heterogeneity signs on NCCT and risk of HE. CI, confidence interval; HE, hematoma expansion; ICH, intracerebral hemorrhage; NCCT, noncontrast computed tomography; OR, odds ratio. [file 6038193.f1.doc]

**ONLINE SUPPLEMENT**

**Heterogeneity signs on noncontrast computed tomography predict intracerebral hematoma expansion: a meta-analysis**

Danfeng Zhang, MD, Jigang Chen, MD, Qiang Xue, MD, Bingying Du, MD, Ya Li, MD, Tao Chen, MD, Ying Jiang, MD, Junyu Wang, MD, Yan Dong, MD, Lijun Hou, MD

**Supplemental Method**

The complete search algorithm for PubMed is as follows.

**PubMed**

((((((((((intracerebral hemorrhage) OR intracranial hemorrhage) OR intracerebral hematoma) OR intracranial hematoma) OR cerebral hemorrhage) OR subdural hemorrhage) OR epidural hemorrhage)) AND (((((hypodensit*) OR density) OR swirl sign) OR blend sign) OR black hole sign))) AND computed tomograph*

| **Supplemental Table I**. Categorization, exposure, original data of included studies. | | | | | | |
| --- | --- | --- | --- | --- | --- | --- |
| First author, year | Sample size | Study endpoints | Exposure | *N* of participants with events | *N* of participants without events | Definition of exposures |
| Subramanian, 2002 | 51 | Poor clinical outcome | Mixed density | 18 | 16 | The CT scans were classified into two categories, predominantly hyperdense, and mixed density. |
|  |  |  | Hyperdensity | 10 | 7 |  |
| Kim, 2008 | 56 | Hospital mortality | Swirl sign | 7 | 6 | It is recognized as an area of low attenuation within an extra-axial hyperattenuating fluid collection. |
|  |  |  | No Swirl sign | 9 | 34 |  |
| Pruthi, 2009 | 109 | Poor outcome | Mixed density | 9 | 28 | A hematoma was considered to be of mixed density when the hypodense areas constituted at least 30% of the hematoma volume. |
|  |  |  | Hyperdensity | 7 | 65 |  |
| Pruthi, 2009 | 109 | Mortality | Mixed density | 8 | 29 | A hematoma was considered to be of mixed density when the hypodense areas constituted at least 30% of the hematoma volume. |
|  |  |  | Hyperdensity | 3 | 69 |  |
| Selariu, 2012 | 203 | Mortality | Swirl sign | 37 | 24 | Swirl sign was defined as region(s) of hypoattenuation or isoattenuation (compared to the attenuation of brain parenchyma) within the hyperattenuated ICH. |
|  |  |  | No swirl sign | 29 | 112 |  |
| Selariu, 2012 | 203 | Poor outcome | Swirl sign | 48 | 11 | Swirl sign was defined as region(s) of hypoattenuation or isoattenuation (compared to the attenuation of brain parenchyma) within the hyperattenuated ICH. |
|  |  |  | No swirl sign | 60 | 69 |  |
| Galbois, 2013 | 72 | Brain death | Swirl sign | 26 | 10 | The swirl sign can be seen on unenhanced CT scans and it represents actively extravasating unclotted fresh blood which is of lower attenuation than clotted blood (typically 50-70 HU) which surrounds it. |
|  |  |  | No swirl sign | 9 | 27 |  |
| Gökçe, 2014 | 45 | Mortality | Swirl sign | 18 | 18 | Swirl sign is defined as a hypoattenuated or isoattenuated region within hyperattenuated ICH. Hypoattenuated or isoattenuated regions vary in shape and are rounded, streak-like, or irregular. |
|  |  |  | No swirl sign | 0 | 9 |  |
| Connor, 2015 | 71 | HE | Swirl sign | 14 | 19 | All hematomas demonstrating enclosed regions that were iso- or hypodense to brain parenchyma were classified as heterogeneous. Heterogeneous. The swirl sign was defined as an intrahematoma region of hypo or isoattenuation compared to the attenuation of brain parenchyma and may be rounded, streak-like, or irregular. |
|  |  |  | No swirl sign | 7 | 31 |  |
| Li, 2015 | 172 | HE | Blend sign | 24 | 5 | Blending of hypoattenuating area and hyperattenuating region with a well-defined margin |
|  |  |  | No blend sign | 37 | 106 |  |
| Boulouis, 2016^a^ | 1029 | HE | Hypodensities | 129 | 191 | Four types of hypodensities: type 1 hypodensity has a brain-like density and distinct margins, type 2 has a brain-like density and indistinct margins, type 3 has an edematous or cerebrospinal fluid-like density, and type 4 has a mixed density with a fluid-fluid level |
|  |  |  | No hypodensities | 95 | 614 |  |
| Boulouis, 2016^b^ | 800 | Unfavorable outcome | Hypodensities | 247 | 57 | Four types of hypodensities: type 1 hypodensity has a brain-like density and distinct margins, type 2 has a brain-like density and indistinct margins, type 3 has an edematous or cerebrospinal fluid-like density, and type 4 has a mixed density with a fluid-fluid level |
|  |  |  | No Hypodensities | 273 | 223 |  |
| Boulouis, 2016^b^ | 800 | Death | Hypodensities | 192 | 112 | Four types of hypodensities: type 1 hypodensity has a brain-like density and distinct margins, type 2 has a brain-like density and indistinct margins, type 3 has an edematous or cerebrospinal fluid-like density, and type 4 has a mixed density with a fluid-fluid level |
|  |  |  | No Hypodensities | 181 | 315 |  |
| Li,  2016 | 206 | HE | Black hole sign | 22 | 8 | The CT black hole sign was defined as (1) relatively hypoattenuated area (black hole) encapsulated within the hyperattenuating hematoma. (2) The black hole could be round, oval, or rod-like but was not connected with the adjacent brain tissue. (3) The relatively hypoattenuated area should have an identifiable border. (4) The hematoma should have at least a 28 Hounsfield unit (HU) difference between the 2 density regions. |
|  |  |  | No black hole sign | 47 | 129 |  |
| Sporns, 2017 | 182 | Secondary neurological deterioration | Blend sign | 31 | 6 | Blending of hypoattenuating area and hyperattenuating region with a well-defined margin |
|  |  |  | No blend sign | 50 | 95 |  |
| Yu,  2017 | 129 | HE | Black hole sign | 14 | 15 | (1) relatively hypoattenuated area (black hole) encapsulated within the hyperattenuating hematoma; (2) the black hole could be round, oval, or rod-like but was not connected with the adjacent brain tissue; (3) the relatively hypoattenuated area should have an identifiable border; (4) the hematoma should have at least a 28 Hounsfield unit (HU) difference between the two density regions |
|  |  |  | No black hole sign | 18 | 82 |  |
| Zheng, 2017 | 115 | HE | Blend sign | 12 | 10 | (1) there is blending of the relatively hypoattenuating area with the adjacent hyperattenuating region within a hematoma; (2) there is a well-defined margin between the hypoattenuating area and the adjacent hyperattenuating region that is easily recognized by the naked eye; (3) the hematoma should have at least an 18 HU difference between the 2 density regions; and (4) the relatively hypoattenuating area is not encapsulated by the hyperattenuating region |
|  |  |  | No blend sign | 16 | 77 |  |
| Abbreviations: CT, computed tomography; HE, hematoma expansion; ICH, intracerebral hemorrhage. | | | | | | |

| **Supplementary Table II.** Quality scores of included studies using Newcastle-Ottawa Scale (maximum score of 9) | | | | | | | | | | | | | | | | |
| --- | --- | --- | --- | --- | --- | --- | --- | --- | --- | --- | --- | --- | --- | --- | --- | --- |
| cohort studies | | | | | | | | | | | | | | | | |
|  | Selection | | | | | | | Comparability | | outcome | | | | | | |
| Reference | Representativeness of the exposed cohort | | | Selection of  the non-exposed cohort | Ascertainment of exposure factors | Demonstration that HE was not present at start of study | | Comparability on the basis of the design or analysis | | | Assessment of outcome | | Adequate follow-up duration (>24 hours) | | Adequate follow-up rate (>80%) | Overall quality |
| Li 2016 | 1 | | | 1 | 1 | 1 | | 2 | | | 1 | | 1 | | 1 | 9 |
| Boulouis, 2016^a^ | 1 | | | 1 | 1 | 1 | | 2 | | | 1 | | 1 | | 1 | 9 |
| Boulouis 2016^b^ | 1 | | | 1 | 1 | 1 | | 2 | | | 1 | | 1 | | 1 | 9 |
| Zheng 2017 | 1 | | | 1 | 1 | 1 | | 2 | | | 1 | | 1 | | 1 | 9 |
| case-control studies | | | | | | | | | | | | | | | | |
|  | | Selection | | | | | | Comparability | outcome | | | | | | | |
| Reference | | Adequate definition of cases | Representativeness of cases | | Selection of controls | | Definition of controls | Comparability on the basis of the design or analysis | Ascertainment of exposure | | | Same method of ascertainment for cases and controls | | Non-response rate (<20%) | | Overall quality |
| Subramanian 2002 | | 1 | 1 | | 1 | | 1 | 0 | 1 | | | 1 | | 1 | | 7 |
| Kim 2008 | | 1 | 1 | | 1 | | 1 | 2 | 1 | | | 1 | | 1 | | 9 |
| Pruthi 2009 | | 1 | 1 | | 1 | | 1 | 2 | 1 | | | 1 | | 1 | | 9 |
| Selariu 2012 | | 1 | 1 | | 1 | | 1 | 2 | 1 | | | 1 | | 1 | | 9 |
| Galbois 2013 | | 1 | 1 | | 1 | | 1 | 2 | 1 | | | 1 | | 1 | | 9 |
| Gökçe 2014 | | 1 | 0 | | 1 | | 1 | 0 | 1 | | | 1 | | 1 | | 6 |
| Connor 2015 | | 1 | 1 | | 1 | | 1 | 2 | 1 | | | 1 | | 1 | | 9 |
| Li 2015 | | 1 | 1 | | 1 | | 1 | 2 | 1 | | | 1 | | 1 | | 9 |
| Yu 2017 | | 1 | 1 | | 1 | | 1 | 2 | 1 | | | 1 | | 1 | | 9 |
| Sporns 2017 | | 1 | 1 | | 1 | | 1 | 2 | 1 | | | 1 | | 1 | | 9 |

0=”No”, “Unable to determine” or “Not available”.

**Supplementary Table III. Subgroup analyses of heterogeneity signs on NCCT and risk of HE.**

| Heterogeneity signs and HE | | | | | | | | | | | | | | |
| --- | --- | --- | --- | --- | --- | --- | --- | --- | --- | --- | --- | --- | --- | --- |
| Subgroups | | | No. of studies | | | OR (95% CI) | | | | *P* for test | *I^2^*(%) | *P* for heterogeneity | | |
| Study design | | |  | | |  | | | |  |  |  | | |
| Cohort study | | | 2 | | | 4.90 [3.16, 7.58] | | | | <0.01 | 25 | 0.25 | | |
| Case-control | | | 4 | | | 5.71 [3.16, 10.32] | | | | <0.01 | 30 | 0.23 | | |
| Sample size | | |  | | |  | | | |  |  |  | | |
| <150 | | | 3 | | | 4.36 [2.48, 7.66] | | | | <0.01 | 0 | 0.74 | | |
| >150 | | | 3 | | | 6.69 [3.41, 13.11] | | | | <0.01 | 62 | 0.07 | | |
| Time interval from symptom onset to CT (hours) | | |  | | |  | | | |  |  |  | | |
| <6 | | | 4 | | | 6.86 [4.27, 11.02] | | | | <0.01 | 1 | 0.39 | | |
| >6 | | | 2 | | | 4.27 [3.17, 5.75] | | | | <0.01 | 0 | 0.61 | | |
| Heterogeneity signs and mortality | | | | | | | | Heterogeneity signs and poor outcome | | | | | | |
| Subgroups | No. of studies | OR (95% CI) | | *P* for test | *I^2^*(%) | | P for heterogeneity | No. of studies | OR (95% CI) | | *P* for test | | *I^2^*(%) | *P* for heterogeneity |
| Study design |  |  | |  |  | |  |  |  | |  | |  |  |
| Cohort study | 1 | 2.98 [2.22, 4.01] | | <0.01 | - | | - | 1 | 3.54 [2.52, 4.96] | | <0.01 | | - | - |
| Case-control | 5 | 6.29 [3.91, 10.10] | | <0.01 | 0 | | 0.90 | 4 | 3.48 [1.35, 8.98] | | 0.01 | | 74 | 0.01 |
| Sample size |  |  | |  |  | |  |  |  | |  | |  |  |
| <150 | 4 | 6.67 [3.35, 13.26] | | <0.01 | 0 | | 0.80 | 2 | 1.57 [0.42, 5.78] | | **0.50** | | 63 | 0.10 |
| >150 | 2 | 3.95 [2.03, 7.69] | | <0.01 | 72 | | 0.06 | 3 | 4.89 [2.83, 8.44] | | <0.01 | | 54 | 0.11 |
| Mechanism of ICH |  |  | |  |  | |  |  |  | |  | |  |  |
| Primary ICH | 4 | 4.41 [2.67, 7.29] | | <0.01 | 50 | | 0.11 | 2 | 4.89 [2.83, 8.44] | | <0.01 | | 54 | 0.11 |
| Secondary ICH | 2 | 7.78 [2.21, 27.42] | | 0.01 | 0 | | 0.49 | 3 | 1.57 [0.42, 5.78] | | **0.50** | | 63 | 0.10 |

Abbreviation: CI, confidence interval; HE, hematoma expansion; ICH, intracerebral hemorrhage; NCCT, noncontrast computed tomography; OR, odds ratio.
